# Social Symptoms of Parkinson's Disease

**DOI:** 10.1155/2020/8846544

**Published:** 2020-12-31

**Authors:** Margaret T. M. Prenger, Racheal Madray, Kathryne Van Hedger, Mimma Anello, Penny A. MacDonald

**Affiliations:** ^1^Department of Neuroscience, Schulich School of Medicine & Dentistry, Western University, London, ON, Canada; ^2^Brain and Mind Institute, Western University, London, ON, Canada; ^3^Clinical Neurological Sciences, Schulich School of Medicine & Dentistry, Western University, London, ON, Canada; ^4^BrainsCAN, Western University, London, ON, Canada

## Abstract

Parkinson's disease (PD) is typically well recognized by its characteristic motor symptoms (e.g., bradykinesia, rigidity, and tremor). The cognitive symptoms of PD are increasingly being acknowledged by clinicians and researchers alike. However, PD also involves a host of emotional and communicative changes which can cause major disruptions to social functioning. These incude problems producing emotional facial expressions (i.e., facial masking) and emotional speech (i.e., dysarthria), as well as difficulties recognizing the verbal and nonverbal emotional cues of others. These social symptoms of PD can result in severe negative social consequences, including stigma, dehumanization, and loneliness, which might affect quality of life to an even greater extent than more well-recognized motor or cognitive symptoms. It is, therefore, imperative that researchers and clinicans become aware of these potential social symptoms and their negative effects, in order to properly investigate and manage the socioemotional aspects of PD. This narrative review provides an examination of the current research surrounding some of the most common social symptoms of PD and their related social consequences and argues that proactively and adequately addressing these issues might improve disease outcomes.

## 1. Introduction

Parkinson's disease (PD) is a neurodegenerative movement disorder characterized by hallmark motor symptoms (e.g., tremor, bradykinesia, and rigidity [1]) brought about by progressive loss of dopaminergic neurons in the substantia nigra pars compacta [2, 3]. PD is currently estimated to affect upwards of 6 million individuals worldwide [4], with prevalence rates expected to double in the coming years [5, 6]. There is currently no cure for PD, though motor symptoms are often well controlled by dopaminergic therapies such as levodopa (i.e., dopamine precursor) and dopamine agonists. Unfortunately, these medications require higher dosages over time to achieve the same level of control over motor symptoms [7].

In addition to the hallmark motor symptoms of PD, researchers have come to appreciate cognitive symptoms that also affect PD patients. These include disruptions to executive processes such as learning and decision making, which are differentially impacted by dopaminergic therapies [8]. For example, compared to PD patients tested OFF medication, PD patients ON medication are better at selecting previously learned responses, but have a harder time learning stimulus-response associations in the first place [9, 10]. Similarly, dopaminergic medication appears to improve deficits in task switching in PD patients, but worsens performance on probabilistic reversal learning tasks [11]. Ongoing research is still uncovering the complex nature of cognitive symptoms of PD.

It has also become clear that PD involves a variety of social symptoms, although these are not as well understood as the motor or cognitive symptoms. In particular, PD patients experience disruptions in emotional expression [12, 13], recognizing others' expressions [14], as well as emotional speech production [15] and perception [16, 17]. These social problems greatly impact patients and their families and can ultimately reduce quality of life [18].

Although the broad nature of these topics prevents us from conducting a systematic review, we hope to provide a general overview of these interconnected areas by highlighting some pertinent experiments and synthesizing ideas across studies. In this narrative review we, therefore, summarize studies that examine the social communication changes that can occur in PD, identify some of the negative social consequences that can arise as a result, and argue that greater awareness of these changes might help improve disease management.

## 2. Social Communication Challenges in PD

### 2.1. Facial Masking

One of the most salient emotional symptoms experienced by PD patients is the reduced ability to spontaneously display emotional facial expressions. This symptom, often referred to as facial masking, is attributed to bradykinesia of the muscles required for facial expressions. Masking often occurs bilaterally, despite the fact that PD typically manifests with worse motor symptoms on one side of the body [19]. Importantly, PD patients with masking do not undergo a decrease in their ability to experience emotional feelings [20]. In other words, these patients are simply unable to display their internally felt emotions via facial expressions. Patients with PD have deemed this inability to accurately express internal emotions as a factor that decreases social wellbeing [21].

In particular, PD patients tend to demonstrate fewer, and less realistic, smiles than healthy controls [22]. Simons et al. [23] demonstrated this quite dramatically by observing reactions when participants were given a surprise gift at the end of the study. Only 73% of PD patients smiled upon receiving the gift compared to 84% of healthy controls, and a much smaller proportion (36%) of PD patients produced a genuine (i.e., Duchenne) smile. The majority produced non-Duchenne smiles, marked by an absence of wrinkling at the corners of the eyes [24]. Duchenne smiles can elicit empathy and feelings of pleasure from observers [25] and, thus, serve a functional purpose. Losing the ability to produce Duchenne smiles in PD can, therefore, have negative social consequences such as the appearance that a patient is cold and withdrawn [22].

Deficits in the spontaneous expression of other emotions have also been observed in PD patients. For example, using video-based automatic scoring algorithms, Bandini et al. [26] suggest that PD patients are especially impaired at spontaneously imitating anger and disgust. However, the patient group in the study did not have as much difficulty voluntarily producing an angry emotional expression when asked to do so by an experimenter. Interestingly, some studies have shown that intentional emotional displays can also be affected by facial masking. Generally, these results suggest that the basic emotions (i.e., happiness, anger, disgust, fear, sadness, and surprise) are all affected equally by facial masking in PD [27, 28].

In addition to facial masking, PD patients are less able to modulate their emotional expressions. In an experiment where spontaneous facial expressions were elicited after smelling pleasant and unpleasant odours, PD patients were less able to produce a requested expression that was incongruent with the valence of the smelled odour [23]. Problems with cognitive flexibility could underly this phenomenon, as PD patients have shown difficulties switching from a salient, automatic response to a response that is less habitual [29–32]. In social interactions, it can be very beneficial to modulate certain facial expressions depending on the context. For example, disguising one's frustrations in a work meeting with a smile could help maintain a positive relationship with coworkers. For PD patients, difficulty modulating facial expressions could cause friction in social relationships which can lead to negative consequences such as increased risk of depression [33].

### 2.2. Emotion Recognition Problems

Additionally, some PD patients experience difficulty recognizing the emotional expressions of others, a phenomenon which has been widely documented and is controversial [14]. Research in this area tends to offer inconsistent conclusions, with some studies finding global emotion recognition problems [34–36], some reporting emotion-specific recognition problems [37–39], and others reporting no deficit at all [40–42]. These differences are likely due to several factors, including methodological differences (e.g., lack of consistency in the type of emotion recognition test and/or stimuli) and heterogeneity in PD patient samples (e.g., differences in disease severity and symptom profiles). More research is needed to clarify the nature of potential deficits in emotion recognition among PD patients.

Furthermore, there is limited evidence on whether dopaminergic therapy has any effect on emotion recognition. Only one study [43] tested PD patients ON and OFF their prescribed medication. Using a forced-choice recognition test with prototypical basic emotion photographs as stimuli (i.e., the Ekman 60-Faces test [44]), the authors demonstrated that PD patients were generally worse than controls at recognizing emotions, particularly when tested in the OFF state. Although limited, some evidence suggests that the administration of levodopa in healthy participants decreases amygdala activation during emotion perception and recognition, but this was not associated with measurable decreases in emotion recognition accuracy [45, 46]. Taken together, these studies indicate that dopaminergic medication indeed affects emotion recognition abilities, and this should be a considered by future studies of emotion recognition in PD.

Interestingly, there is some evidence that the problems PD patients have in recognizing others' emotions are related to their own facial masking [14, 34, 47]. According to embodied simulation theory, mimicking an expression provides sensorimotor cues which act as feedback to the brain to aid in emotion recognition [48, 49]. Studies with healthy participants have shown that disrupting facial mimicry can impair emotion recognition [50, 51]. For example, when facial mimicry is blocked, such as through facial manipulations including chewing gum or biting a pencil [51] or chemical interference such as botulinum toxin [50, 52], emotion recognition accuracy tends to decrease. If masking decreases PD patients' ability to mimic the expression of others, this might directly affect their emotion recognition abilities. Indeed, facial mimicry deficits have also been demonstrated in PD [33, 53], although only one study has linked this deficit to reduced emotion recognition accuracy [34].

### 2.3. Dysarthria

Some PD patients also experience disruptions to their normal speech patterns, such as dysarthria. Dysarthria involves producing speech which is grammatically and syntactically correct, but that is marked by abnormal rhythm, harsh voice, inappropriate pauses, and prosodic loss [54]. PD patients with prosodic loss have monotonous, nonemotional speech [55, 56]. Interestingly, dysarthria might only manifest during spontaneous speech, as opposed to other communication methods such as singing or reading [57]. The effect of dopaminergic medication on dysarthria and prosodic loss in PD remains relatively unclear. Generally, it appears that while dopaminergic medication (i.e., levodopa) might improve some aspects of speech (e.g., vowel articulation [58]), speech intelligibility and prosody are rarely affected [58–61]. However, some researchers have found improvements in the ON medication state [62, 63]. Nevertheless, these disruptions in spontaneous speech have been identified as a distressing factor in the lives of patients [64] likely because of the burden they place on social communication.

PD patients with dysarthria have demonstrated negative self-perception, expressing frustration as they feel less competent in their communication abilities and less able to communicate what they intend to say [65]. Importantly, this was found to be independent of actual intelligibility of speech, highlighting that negative self-perceptions can arise regardless of the severity of the communication problem [65]. Communication in PD might also be affected by cognitive changes. For example, one study identified that many PD patients have to put conscious effort into speaking [66]. This effort can be draining and can cause patients to feel frustrated and embarrassed.

### 2.4. Prosody Identification Deficit

In addition to difficulty producing expressive speech, PD patients also have difficulty identifying the emotional prosody (i.e., pitch, intonation, and rhythm used to convey emotion) of others' speech [17, 67, 68]. Similar to emotion recognition problems, these difficulties have been demonstrated for specific negative prosodic emotions, including fear, anger, and disgust [69], and for global prosody perception [16, 70]. Yet, some studies report no deficit at all [68, 71]. Some researchers suggest that prosody reception deficits only occur in those with later-stage PD accompanied by cognitive impairment [72].

Furthermore, little attention has been paid to the effect of dopaminergic therapy on prosody recognition in PD. In one study, early-stage PD patients demonstrated prosody identification deficits, particularly while ON medication [73]. Another study found no difference in prosody recognition across medication states for both orally medicated PD patients and those who had undergone deep brain stimulation surgery [74]. However, it is clear that methodological differences and sample variability might contribute to the discrepant findings in this area.

### 2.5. Future Directions

The aforementioned verbal and nonverbal behaviors are vital components of communication that provide contextual information during social exchanges. The loss of emotional expression and emotion recognition abilities, as well as changes in emotional speech production and reception, therefore, can have devastating consequences for PD patients and their families. There is a need for a more detailed study of facial and prosodic emotion recognition deficits in this patient population. While some of the discrepant findings might be due to differences in methodology and sample characteristics, others might be because of dopamine replacement therapy. Futures studies are needed to provide clarification.

## 3. Negative Consequences Associated with Social Symptoms of PD

### 3.1. Stigma

Stigma is a negative perception that discredits or devalues an individual within a social or societal context (e.g., people with disabilities and negatively stereotyped racial groups); for an overview, see [75]. Some researchers have argued that stigma exists because it provided an evolutionary advantage via a disease-avoidance mechanism [76, 77]. As Park and colleagues describe [77], this mechanism can operate in response to physical disability cues, even when it is clear that the disability is not the result of a contagious disease. In the present day, rather than promoting group fitness, stigma has primarily negative effects on those who experience it [78]. Moreover, self-stigma can occur in individuals with chronic health conditions and can further amplify the negative effects of public stigma (e.g., psychological distress and reduction of self-worth [79]). Stigma can affect not only how patients are viewed and treated by others around them but also how they view themselves and their expectations about their future social relationships.

Facial masking, in particular, has been researched as a contributing factor to perceptions of stigma in PD. One recent study found that stigma associated with facial masking was the main reason for reduced quality of life in PD patient participants [80]. In fact, stigma was found to be a stronger mediator of the relationship between facial masking and quality of life than depression. This is, perhaps, because observers tend to form negative impressions of those with emotional expression deficits, which has been shown in patients with facial paralysis [81]. This can lead patients to feel misunderstood and can hinder social communication with caregivers and health care workers. The emotional expressions PD patients produce are often mistaken by peers for negative emotions, which results in PD patients being considered less socially desirable, friendly, and attentive [82–84]. As a result, PD patients are less able to make a favourable first impression on peers, particularly during emotional exchanges [85]. This effect tends to be worse for women with PD, perhaps due to social gender norms that suggest women are more emotional [86]. Indeed, Ma et al. [80] found that women tended to experience a greater degree of perceived stigma as a result of facial masking.

Similarly, PD patients with dysarthria have expressed that they experience stigma when communicating in public and worry about the perceptions of others. Listeners of speech recordings rate PD participants as being more unhappy and less friendly than their control counterparts [87]. This is likely due to prosodic loss, as one study indicated that listeners were less able to detect intended emotions such as anger and disgust within PD patient speech recordings compared to that of healthy controls [15]. Unfortunately, the stigma associated with speech problems in PD can also make patients vulnerable to discrimination. One study cited incidences such as being asked not to read aloud in a Bible study group as an example of the negative consequences associated with speech-related stigma [66]. It is, therefore, not surprising that nearly 40% of patients report that communication changes in PD are among their top disease-related concerns [88].

Stigma accompanies many mental and physical disorders, but in the case of PD, it can be particularly detrimental. Perception of stigma is directly related to perceived quality of life for PD patients, even when controlling for disease severity and motor difficulties [89]. As the disease progresses, patients with PD might experience an exacerbation of nonmotor symptoms, in addition to motor symptoms, leading to stigma associated with their communication problems (for review, see [90]). The stigma associated with both nonmotor (communication) deficits and motor symptoms puts these patients at a greater risk for dehumanization, a link that has been demonstrated in other patient populations (e.g., those with mental illnesses) who are perceived as lower in social status due to their disorder [91].

### 3.2. Dehumanization

Dehumanization is categorized by a denial of the capacity for experience (e.g., feelings of pleasure or pain) and/or agency (e.g., making plans or choices) of another person [92]. Dehumanization does not have to be explicit and discriminatory, and it can also occur implicitly based on automatic, unconscious perceptions of others [93].

Unfortunately, dehumanization is a common occurrence in both formal (i.e., with physicians or in a healthcare setting) and informal (i.e., with friends or loved ones) health contexts [94], taking forms such as neglecting patient perspectives or ignoring patients concerns. Although it can have negative effects on patients, dehumanization by healthcare workers might offer these workers some protection from the symptoms of burnout [95]. Patient-centered care approaches have made some headway in combating dehumanization by physicians and healthcare workers; however, more work is needed to overcome dehumanization in informal health-related settings (i.e., by caregivers or even self-dehumanization).

Dehumanization of PD patients in clinical settings can occur for several reasons. Modern healthcare settings require neurologists to assess and discuss all of the different aspects of PD, including motor, cognitive, and social symptoms, during appointments that typically last 30 to 60 minutes at most. To extract important information from patients in a very short amount of time, clinicians often rely on nonverbal communication as a way of assessing how the patient is doing overall [96]. Unfortunately, this strategy is likely to be ineffective with PD patients who experience significant socioemotional or communication deficits. Due to facial masking and dysarthria, it can take PD patients much longer to communicate in social interactions [97], including those with clinicians. In some instances, a caregiver (e.g., spouse and child) will take control of an appointment to provide the clinician with pertinent information more quickly. Although effective at transmitting information, this approach can be detrimental, as the patient might feel as though their voice has not been heard.

Clinicians' negative perceptions of PD patients as a result of social communication challenges can also contribute to dehumanization in healthcare settings. For example, clinicians tend to perceive PD patients with facial masking as more depressed, less intelligent, and less sociable than patients without [98]. Healthcare workers with less experience were particularly guilty of this, labelling PD patients with masking as having a more negative personality [99]. Similarly, clinicians have been found to judge patients with acquired dysarthria as less cognitively competent than those without communication challenges [100]. Awareness of the tendency to dehumanize patients with facial masking and dysarthria is an important first step towards mitigating this issue.

### 3.3. Social Isolation and Loneliness

One major outcome of stigma and dehumanization is social isolation, as insecurity in social spaces and internalized stigma can lead PD patients to avoid social interactions or avoid disclosing information about themselves to others [101]. Repeated social isolation can put one at risk for experiencing loneliness, which is a persistent and debilitating state of perceived social disconnection that can affect mental and physical health. Importantly, feelings of loneliness are distinct from objective assessments of social isolation, although both can negatively affect health outcomes [102]. Cacioppo et al. [103] identified loneliness, in particular, as a risk factor for morbidity and mortality. Moreover, there are clear health-related and cognitive benefits to being well connected to a diverse social network [104].

Unfortunately, rates of loneliness and associated psychological distress can increase with age among older adults [105]. Compared to young and middle-aged adults, older adults typically experience decreasing physiological resilience, and loneliness can further exacerbate their susceptibility to the adverse effects of life stressors [106]. Social isolation and loneliness are critical factors to address in older adults in general, and PD patients and caregivers are faced with additional disease-related challenges that might make them more prone to experiencing these issues.

The social symptoms of PD often directly lead to feelings of loneliness and social isolation. Indeed, one study found that patients with greater facial masking have reported experiencing greater self-reported social exclusion [21]. In another study, over half of PD participants blamed masking specifically for their feelings of social distance between themselves and their partners [107]. Dysarthria can contribute to social withdrawal as well. Feelings of frustration and embarrassment caused by speech difficulties in PD can lead patients to avoid communication opportunities [108, 109]. Moreover, even when PD patients with dysarthria do attempt to communicate, conversation partners tend to dominate [110].

Unfortunately, as patients begin to experience greater feelings of loneliness, they might also experience changes in the healthcare they receive. In one study, clinicians who interacted with elderly patients self-declared that they and their colleagues provided more comprehensive care to the patients who seemed less lonely and who had greater social support [103]. This suggests that experiencing social symptoms in PD can initiate a positive feedback loop in which the negative consequences (e.g., stigma and dehumanization) of these symptoms lead to social withdrawal, which further exacerbates the negative consequences.

The caregivers of PD patients are also at risk for experiencing loneliness and social isolation. Caregiving can be stressful, and across several conditions (e.g., depression, autism, or multiple sclerosis), caregivers are at a greater risk for depression, feelings of general fatigue, and decreases in life satisfaction [111, 112]. For caregivers of PD, those with low self-efficacy in their caregiving abilities are more likely to experience loneliness [113]. However, this can be mitigated by greater social support through daily interactions or support groups [113].

Overall, loneliness might manifest in patients with PD due to the psychosocial and physical consequences that accompany its progression (i.e., decreased emotional expression and physical autonomy). Likewise, caregivers might also experience feelings of loneliness as their caregiving role can limit their ability to maintain engagement with others outside of this role. Fortunately, although loneliness is a multifaceted issue, loneliness is cognitive in nature, and some researchers have suggested that clinicians can intervene by helping patients develop a more positive outlook on the disease and their achievements and challenges [114].

## 4. Clinical Implications

Early identification of socioemotional symptoms is essential for preventing subsequent negative consequences. As one study mentions, delayed intervention can lead to the development of negative coping strategies such as withdrawal and denial [64]. Furthermore, it has been well established that certain symptoms of PD manifest years before the disease is ever diagnosed [115, 116]. Clinicians who are aware of these sometimes “invisible” socioemotional symptoms can incorporate their management into a holistic treatment plan.

There are several tools which can be used to assess the severity of PD-related social symptoms, as well as their related consequences. For example, the Dysarthria Impact Profile (DIP) is a self-assessment tool which evaluates the psychosocial impact of acquired dysarthria [117] and has demonstrated reliability and validity in PD patient samples [118–120]. The 39-Item Parkinson's Disease Questionnaire (39-PDQ [121]) is considered the most well-validated instrument for assessing health-related quality of life in PD by the Movement Disorder Society [122]. It includes items that address communication difficulties, social isolation and loneliness, stigma, and social support and, thus, could be used as an effective measure to assess and monitor PD-related social symptoms and associated negative consequences.

Furthermore, education for patients and their caregivers about socioemotional symptoms and associated consequences can help prepare patients for these potential issues [107]. Moreover, encouraging patients to have an active role in educating their family and friends about their disease can be helpful in reducing social isolation [64, 114].

### 4.1. Management of Social Symptoms in PD

Some PD patients have employed informal strategies to compensate for socioemotional symptoms such as facial masking and dysarthria. For example, patients might turn to other channels of communication (e.g., physical touch, e-mail, and note writing) to more clearly express their emotions [64, 107]. More formal strategies include exercises designed to preserve or amplify patients' communication abilities [107]. For example, the Lee Silverman Voice Treatment (LSVT LOUD [123]) combines vocal and respiratory exercises designed to increase vocal loudness. LSVT LOUD has been shown to improve both dysarthria and facial masking in PD patients [124, 125] and was determined to be one of the most effective treatments for improving pitch in a recent meta-analysis [126]. However, it is essential to tailor treatment recommendations to individual patient needs, especially in light of the fact that certain treatments for emotional and communication symptoms might not be viewed positively by patients. For example, one group of patients indicated that speech drills were not sufficient to address important social aspects of communication [66]. Choral singing treatment appears to be more enjoyable for patients [127] and yields some positive outcomes, such as improvements in mood [128], perhaps due to its highly social nature.

Importantly, it is currently unclear how dopaminergic medication affects social symptoms such as facial masking, dysarthria, and the perception of verbal and nonverbal emotional expression. Despite the lack of evidence in this area, some patients report self-administering extra dopaminergic medication to help alleviate social symptoms such as facial masking [107]. However, this could cause further problems (e.g., dyskinesias) in the long run [129]. As previously mentioned, dopaminergic medication can also have differential effects on the cognitive symptoms of PD, improving some, despite worsening others, and it is possible that the same effect could be observed for social symptoms. Further research must be conducted to investigate whether and how the social symptoms of PD are affected by conventional dopaminergic treatments.

## 5. Conclusions

In conclusion, there is ample evidence that PD involves social consequences, and that the hallmark motor symptoms of PD might further contribute to these issues. Specifically, motor deficits including bradykinesia of the facial muscles and dysarthria can affect patients' ability to express emotions or impact speech during conversations, respectively. The emergence of social symptoms is common and disruptive to patients' lives, as feelings of stigma, dehumanization, and loneliness might lead patients to withdraw from social situations. Even further, these social symptoms might extend to caregivers of patients with PD, as they might also feel lonely due to the nature of their caregiver role and the consequences of stigmatization. In the future, systematic review of topics related to the social symptoms of PD would help to extend and clarify the findings presented in this narrative review.

Unfortunately, the social symptoms of PD are less well recognized and can significantly decrease patients' quality of life. These socioemotional symptoms and consequences of PD are addressable but anticipating potential social issues requires (a) acknowledging and preparing for them before they occur and (b) actively re-examining whether social needs are being adequately met throughout the course of the disease. Fortunately, recent research proposes novel strategies to help address these issues.

## Data Availability

No data were used to support this study.
